# Similarity analysis, synthesis, and bioassay of antibacterial cyclic peptidomimetics

**DOI:** 10.3762/bjoc.8.128

**Published:** 2012-07-24

**Authors:** Workalemahu M Berhanu, Mohamed A Ibrahim, Girinath G Pillai, Alexander A Oliferenko, Levan Khelashvili, Farukh Jabeen, Bushra Mirza, Farzana Latif Ansari, Ihsan ul-Haq, Said A El-Feky, Alan R Katritzky

**Affiliations:** 1 Center for Heterocyclic Compounds, Department of Chemistry, University of Florida, Gainesville, FL 32611-7200, USA; 2 Department of Pharmaceutical Organic Chemistry, Faculty of Pharmacy, Zagazig University, Zagazig-44519, Egypt; 3 Department of Organic Chemistry, College of Pharmacy, Misr University for Science and Technology, Al-Motamayez District, P.O. Box: 77, Egypt; 4 Department of Chemistry, Quaid i Azam University, Islamabad 45320, Pakistan; 5 Department of Biochemistry, Quaid i Azam University, Islamabad 45320, Pakistan; 6 Department of Chemistry, King Abdulaziz University, Jeddah 21589, Saudi Arabia

**Keywords:** antibacterial, cluster analysis, *N*-acylbenzotriazoles, peptidomimetics, similarity

## Abstract

The chemical similarity of antibacterial cyclic peptides and peptidomimetics was studied in order to identify new promising cyclic scaffolds. A large descriptor space coupled with cluster analysis was employed to digitize known antibacterial structures and to gauge the potential of new peptidomimetic macrocycles, which were conveniently synthesized by acylbenzotriazole methodology. Some of the synthesized compounds were tested against an array of microorganisms and showed antibacterial activity against *Bordetella bronchistepica*, *Micrococcus luteus*, and *Salmonella typhimurium*.

## Introduction

Diverse cyclic peptides, both natural and synthesized, are potent antibiotics [1–3]. Gramicidin S, vancomycine, bacitracin, polymixin B, colistin, valinomycin, actinomycin, and many more, have been tested and used clinically as antimicrobial and antifungal agents: It is believed that cyclic peptides are more stable to proteolysis due to the lack of free N- and C-termini, as well as reduced conformational freedom. Stability can also be achieved by modifying peptides into “peptidomimetics” that mimic and/or stabilize the secondary structure that modifies associated biological processes, thus affording opportunities for drug design and development.

One strategy to create peptidomimetics couples a small-molecule scaffold with a peptide. Such scaffolds include aromatic rings or heterocycles, which may be positioned in the interior of the peptide chain [4–5] or at the C- [6–8] or N-terminus [9]. Many reports describe the successful use of heterocycles as peptide-bond surrogates or as potential protein-recognition motifs to achieve superior potency in biological assays [10–14].

Pyridines are well-established as important heterocycles in medicinally and biologically active compounds and also as important structural elements. Pyridine scaffolds possess important antiviral [15], anti-inflammatory [16–17], anticonvulsant [18], antibacterial [19], and antitumor pharmacological activities [20–21].

Chiral macrocyclic ligands have found wide application in asymmetric synthesis and enantiomeric recognition [22–23]. Incorporation of amino acids in abiotic anion receptors can lead to systems that mimic the anion coordination properties of anion-binding proteins [24]. Introduction of cysteine subunits into a macrocycle facilitates receptor synthesis and allows control of the relative direction of the two chains attached to the cysteine residue. For example, one of the early cysteine-containing macrocycles was designed to mimic the cation binding ability of valinomycin [24].

Numerous literature approaches to the synthesis of 17-, 18- and 24-membered rings utilize: (i) acyl chlorides, (ii) active esters and (iii) coupling reagents. However, some of the published literature methods involve the utilization of complex procedures, protection/deprotection strategies, harsh reaction conditions, and long reaction times; provide only low yields; and are plagued with difficulties associated with product purification [25–29]. According to the literature [30–31], most antimicrobial cyclic peptides are active in their cationic form. Cationic peptides are considered advantageous, because they adhere better to the outer anionic parts of phospholipid membranes.

Although anionic peptides are less common in clinical practice, they are gaining momentum, because natural anionic peptides are a part of the innate immune system of living organisms (located in human, ovine, and bovine lungs) and also serve as immunomodulators [32]. The activity of anionic peptides can be enhanced when associated with zinc cations and/or surfactants [30–32]. Anionic peptides bind with lysozyme, an important cationic hydrolytic enzyme. It is believed that the lysozyme opens the cell wall, “allowing a small anionic peptide to penetrate”. Topical administration of such anionic peptides is possible, for example in the form of nasal or throat sprays but, at least one anionic peptide, daptomycin, is administered intravenously.

The abundance of natural and synthetic antimicrobial peptides and peptidomimetics of varying structure and activity reported raises the question of how their similarity or dissimilarity can be quantified and how synthetic efforts can be focused towards novel active compounds. It would be beneficial to be able to draw practical conclusions (even if approximate) based solely on the chemical structures of existing agents. In the case of cyclic peptides, the complex cyclomatic structure, involving smaller rings and heteroatoms, as well as a limited conformational flexibility, makes it difficult to classify or compare such compounds by their ring sizes. Fortunately, chemical informatics provides formalized procedures for comparing chemical compounds based on composition and structure. While the principle of molecular similarity is quite simple, that is, “similar compounds have similar properties”, the unambiguous and automated implementation of this principle needed to be backed with a solid mathematical background; this has been accomplished and has made it possible to develop efficient similarity measures [33]. The simplest similarity measure of two molecules would be the Euclidian distance between them in a descriptor space, which is an abstract *n*-dimensional space spanned by *n* molecular descriptors, or parameters. More elaborate measures have been developed, such as the Tanimoto coefficient or the Tversky index [34]. Although a well-established concept, molecular similarity has previously been used almost entirely in the design of small-molecule drugs, with very few applications in other chemical disciplines. To the best of our knowledge, despite their promise of consistent analysis and comparison, the principles of molecular similarity have never been used in the design of peptides.

The aim of the present work is thus three-fold: (i) To identify promising peptidomimetic scaffolds by using molecular similarity; (ii) to design a range of cyclic pyridine-containing peptidomimetics capable of facile synthesis; and (iii) to synthesize and biotest promising candidates. A synthetic route seems highly relevant, since *N*-acylbenzotriazoles have been reported elsewhere [35–37] to be stable, easy-to-handle acylating agents and have found numerous applications for advantageous N-, O-, C- and S-acylations.

## Results and Discussion

### Similarity analysis

Our literature search identified the thirty three cyclic peptides and peptidomimetic structures given in Table 1. All the minimum inhibition constants (MIC) are pertinent to *Staphylococcus aureus*. These include (i) one 12-membered ring cyclic tetrapeptide, (ii) two 14-membered ring peptidomimetics each with two peptide links, (iii) two 18-membered ring peptidomimetics each with five peptide links, and (iv) twenty seven 16-membered ring pentapeptides. Table 1 demonstrates that compounds of group (iv) show the best antibacterial activity, with compounds **24** and **27** being the most active. The side-chain substitution clearly controls the antibacterial activity, as the cyclic scaffold is identical. The 18-membered ring scaffold **37**, accessible through the benzotriazole route, provides an excellent opportunity for introducing substituents economically, through the proper choice of the dicarboxylic starting materials. While scaffold **37** and the 16-membered ring scaffolds described in Table 1 seem to be visually different, we undertook analysis of their proximity in a multidimensional descriptor space to afford a more rigorous comparison. Such analysis takes into account not only structural similarity features, but also electronic, charge-distribution, and hydrogen-bond characteristics. We generated such a multidimensional descriptor space for all structures in Table 1 as well as for structures **37a**–**c**, using CODESSA-Pro software [38]. Clustering of the structures in such a multidimensional space is the natural choice for similarity analysis.

**Table 1:** Structures of cyclic peptides with their MIC values.

Structure	Cluster number	Observed MIC (μg/mL)	Ref.

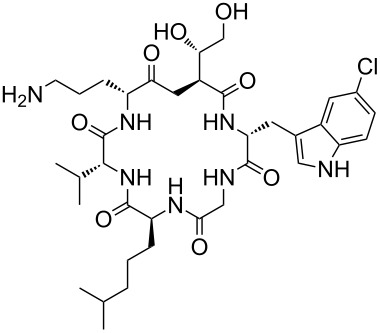 **1**	3	8.0	[39]
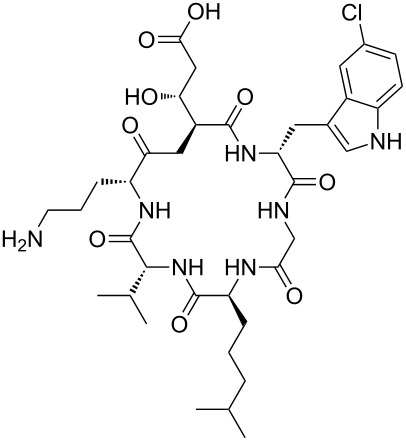 **2**	2	16	[39]
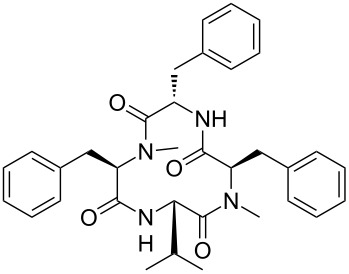 **3**	3	--	[40]
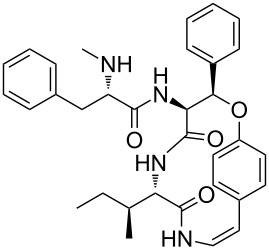 **4**	3	3.12	[41]
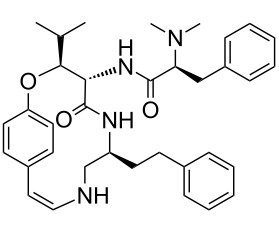 **5**	3	3.12	[42]
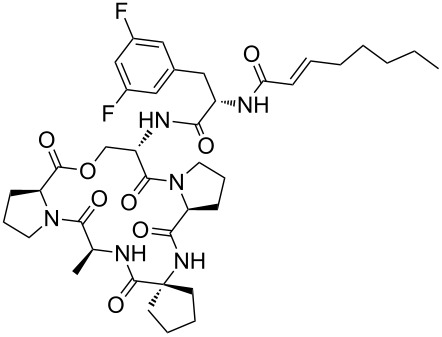 **6**	2	0.50	[43–44]
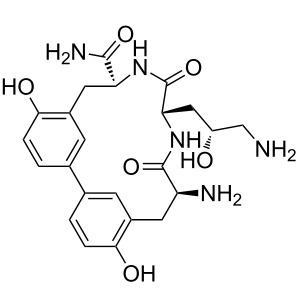 **7**	3	1.5	[44]
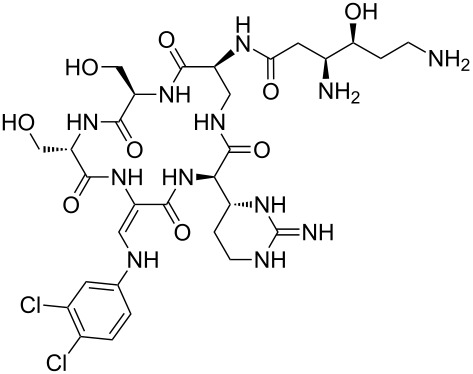 **8**	1	12.5	[45]
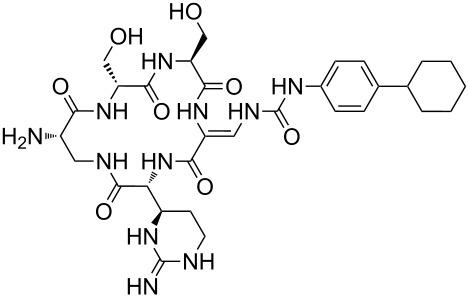 **9**	2	50	[45]
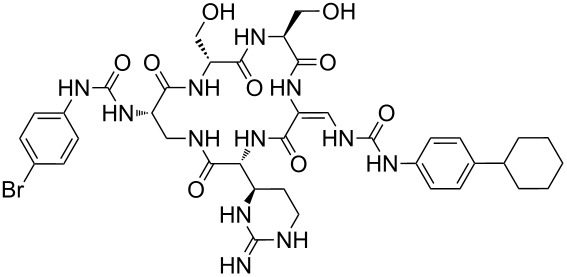 **10**	1	12.5	[45]
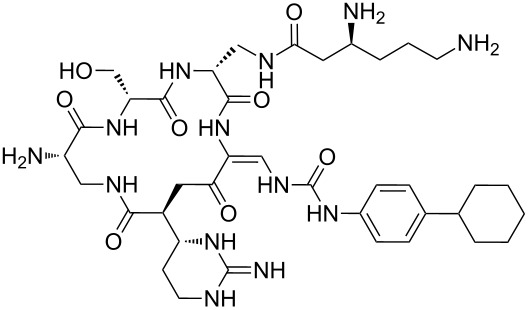 **11**	2	1.56	[45]
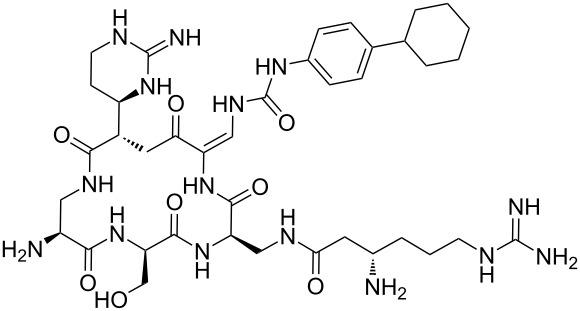 **12**	1	1.56	[45]
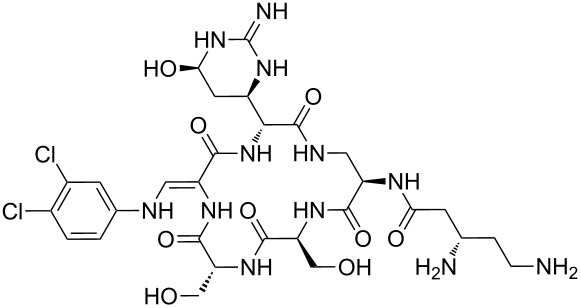 **13**	2	6.25	[46]
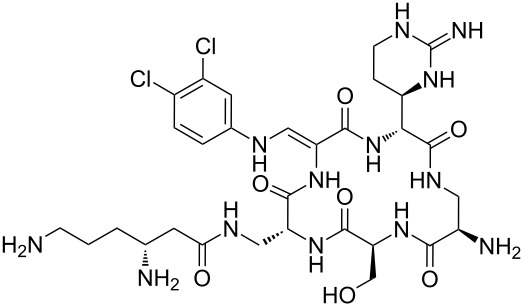 **14**	2	1.56	[46]
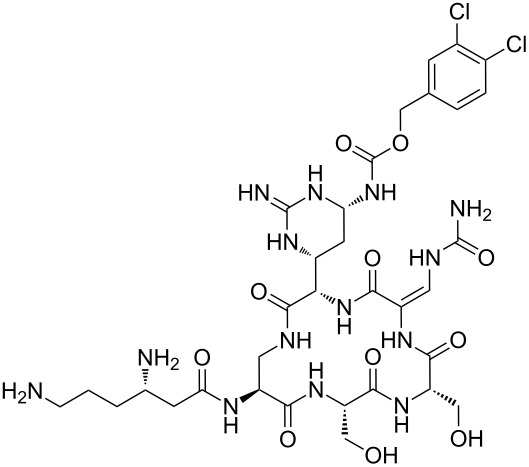 **15**	2	3.12	[47]
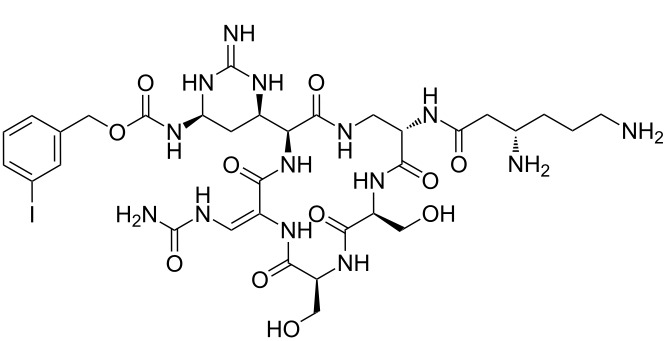 **16**	2	12.5	[47]
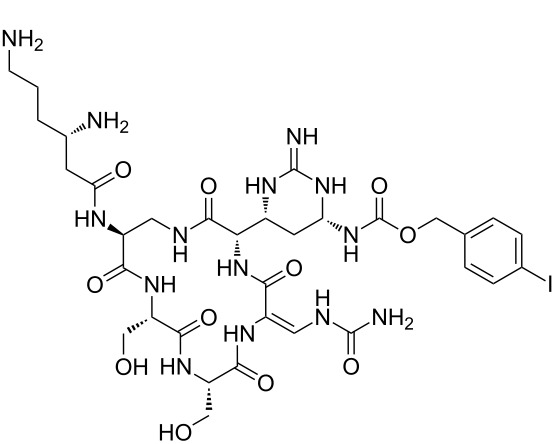 **17**	2	12.5	[47]
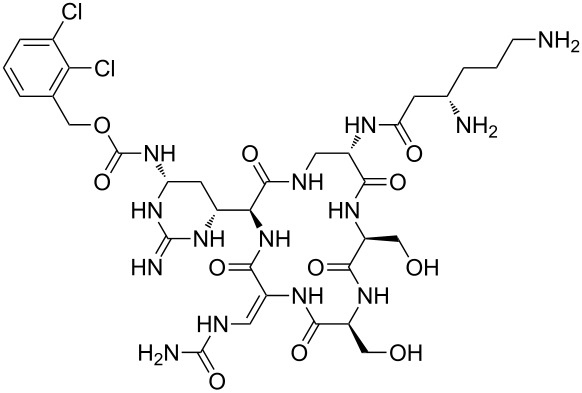 **18**	2	12.5	[47]
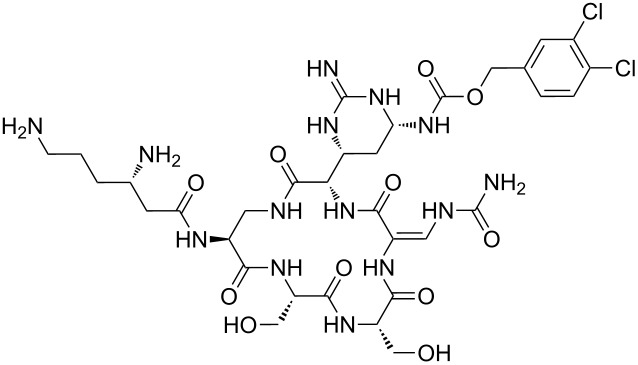 **19**	2	12.5	[47]
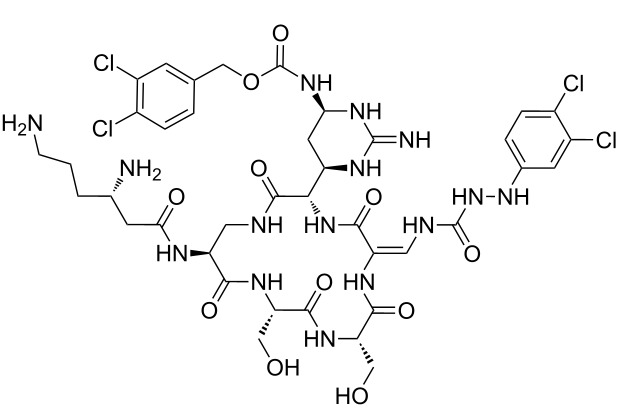 **20**	1	25.0	[47]
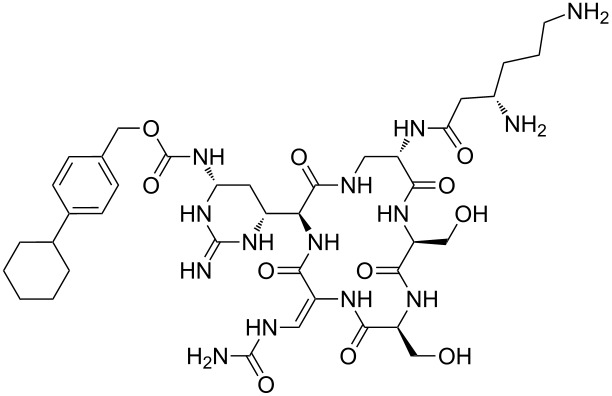 **21**	2	25.0	[47]
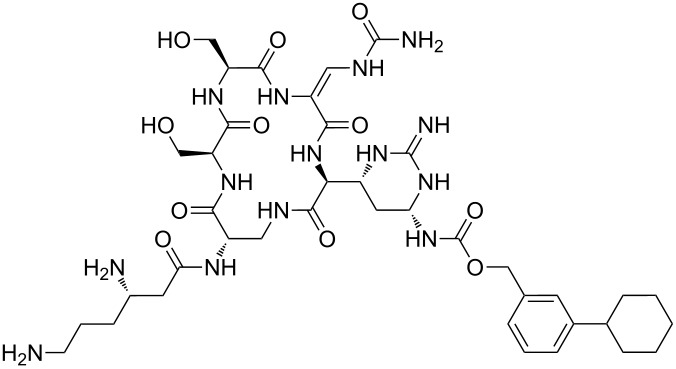 **22**	2	12.5	[47]
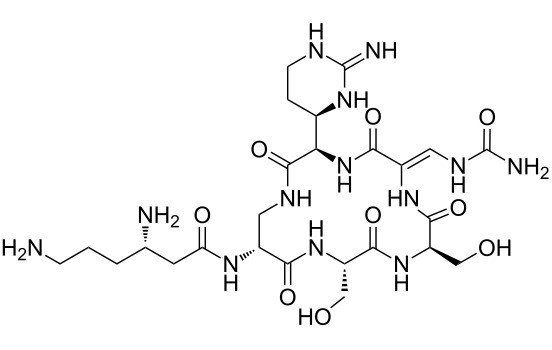 **23**	2	12.5	[47]
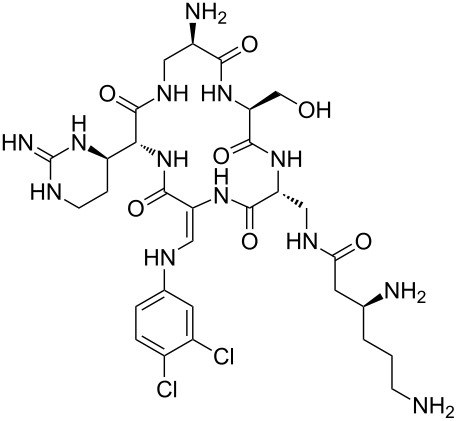 **24**	2	0.78	[48]
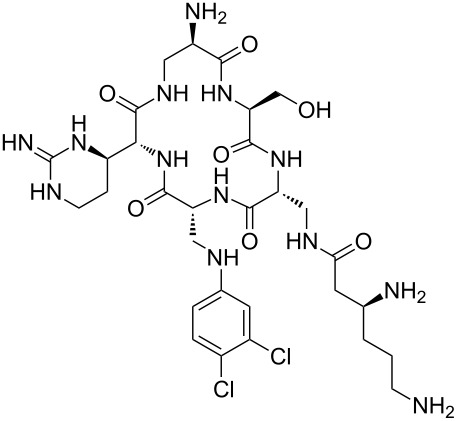 **25**	2	6.25	[48]
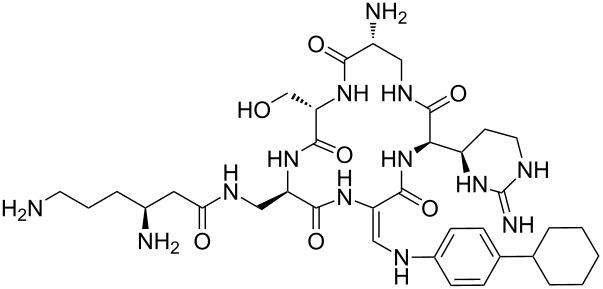 **26**	1	1.56	[48]
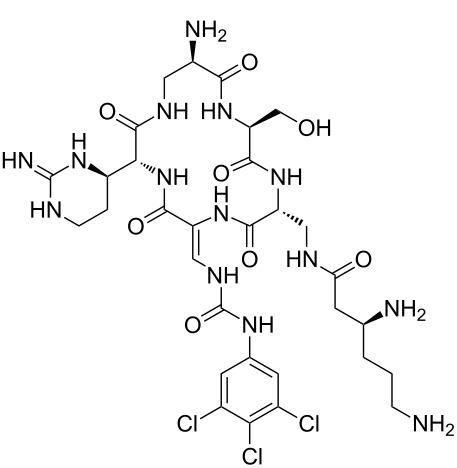 **27**	2	0.780	[48]
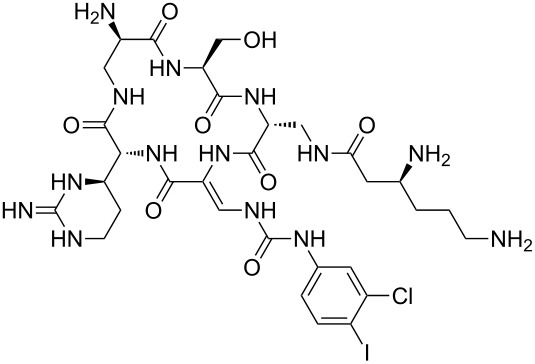 **28**	2	3.12	[48]
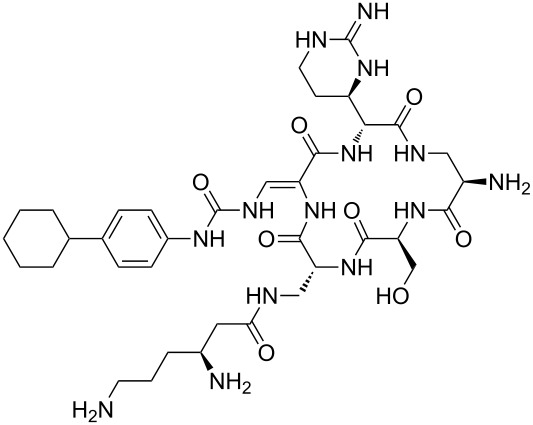 **29**	2	1.56	[48]
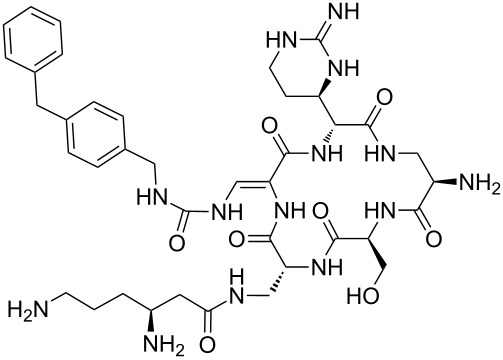 **30**	2	1.56	[48]
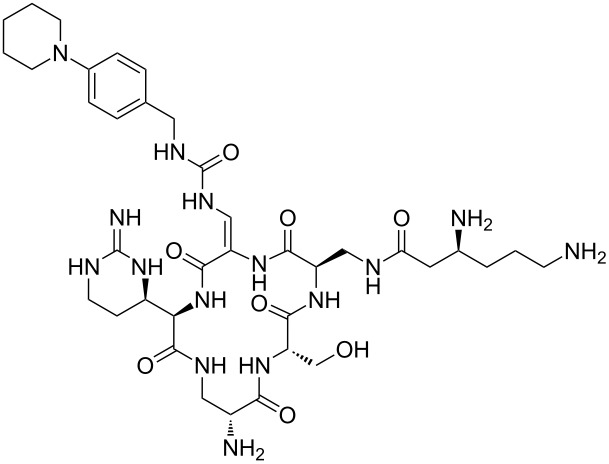 **31**	2	3.12	[48]
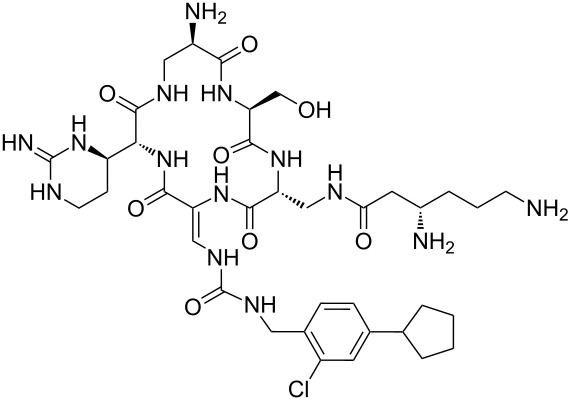 **32**	2	3.12	[48]
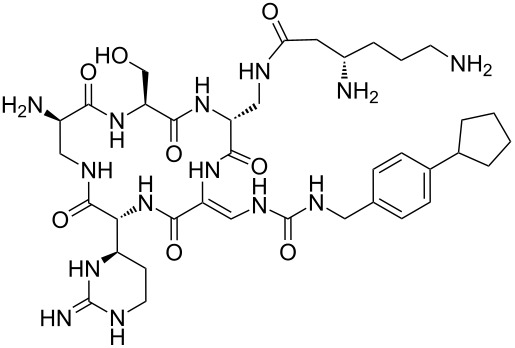 **33**	2	3.12	[48]

### Clustering

Clustering is a widely used technique that has found application in the selection of compounds for screening, analysis of substructure search output, and the prediction of molecular properties [49]. The “*k*-means method” implemented in Statistica software is effective in clustering results for many practical applications, and was used to classify the current data set. All types of descriptors are calculated by using the CODESSA Pro program and the descriptor matrix is generated, which served as an input for the cluster-analysis module in STATISTICA software. The use of descriptors in this study can give differing clustering patterns [50]. The first step in the structure clustering is the generation of a matrix containing similarity values of descriptors for all pairs of compounds. Further, the matrix is converted to a hierarchical clustering tree by using single-linkage amalgamation and Euclidian distance measure. The single-linkage approach assigns sequential cluster labels to nearest-neighbor clusters. Then a series of clustering experiments was carried out with different cutoffs on the descriptor dependence, to clearly establish whether parameters produce realistic improvements in the quality of the results or not [51].

**Figure 1:**
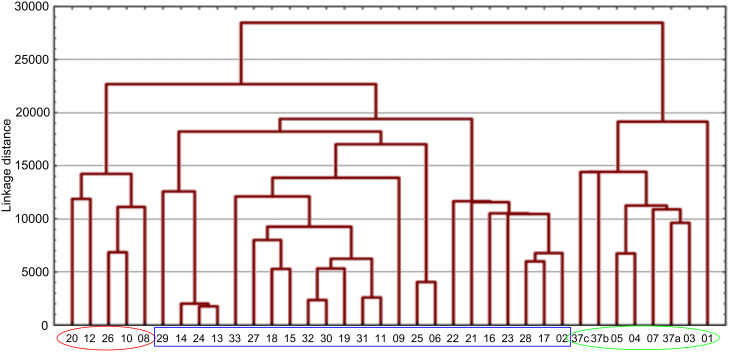
Molecular-descriptor-based cluster analysis; single-linkage Euclidean distances. Clustering of compounds in the descriptor space; a two-dimensional representation of chemical space being partitioned into clusters of similar compounds based on descriptors using a top-down (hierarchical) clustering method. Structures for compounds **37a**–**37c** are shown in Scheme 2. (Axis X denotes compounds and axis Y denotes linkage distance); the red oval, blue rectangle, and green oval represent cluster 1, cluster 2, and cluster 3, respectively.

Analysis of the membership functions shows that the clusters are well-defined with each compound typically having one membership function, as shown in Figure 1 and Table 1. The cluster analysis results demonstrate that most of the studied compounds are clustered clusters. The peptidomimetics **37a**–**c** proposed for synthesis are located in cluster 1. Cluster 1 mainly consists of both cyclotetra- and cyclopentapeptides that are similar in ring size to **37a**–**c**; this cluster also includes those cyclic peptides that are highly potent as antibacterial agents (smallest MIC values). Clusters 1 and 2 contain structurally more diverse peptides. Thus, the majority of 12-membered ring-cyclic compounds were assigned to cluster 1, while the more branched structures with longer side chains were found in cluster 2. These assignments are chemically meaningful and thus bear witness to the validity of the clusterization method used.

The proximity of the cyclic peptidomimetics under study (**37a**–**c**) and the existing antibacterial cyclic peptides in such a rich descriptor space, in which all major structural, electronic, and intermolecular-interaction features are taken into account, attests to the feasibility of using the 17- and 18-membered ring scaffolds **37** as a main building block for designing novel antibacterial agents. This, along with the facile and economic synthetic route, renders scaffold **37** as a promising platform for further rational drug design.

### Synthesis

**Scheme 1:**
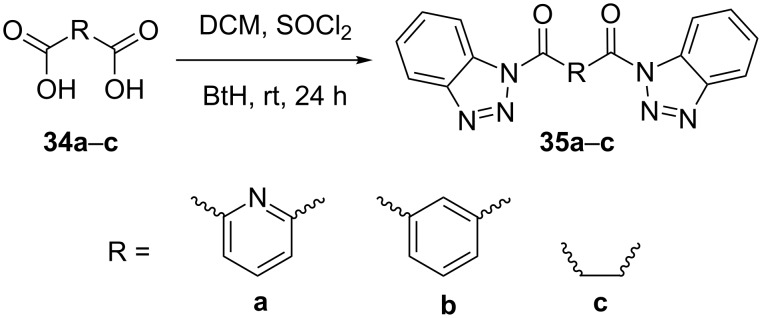
Preparation of dicarboxylic benzotriazole derivatives.

Treatment of dicarboxylic acids **34a**–**c** by a standard method [36] using thionyl chloride and 1*H*-benzotriazole gave the corresponding benzotriazole derivatives in 37–54% yield (Scheme 1, see Supporting Information File 1 for experimental details).

**Scheme 2:**
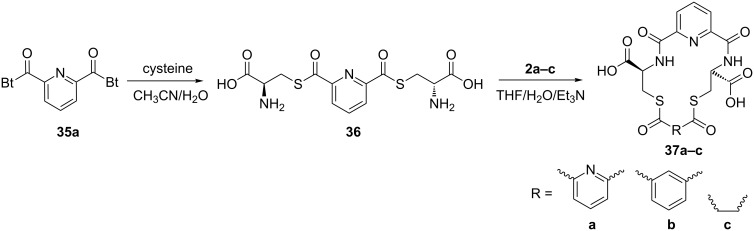
Preparation of pyridine-based cysteine-containing macrocycles.

The methodology used for the regioselective syntheses of *S*- and *N*-acylcysteines was developed recently in our group by using *N*-acylbenzotriazoles under mild reaction conditions [37]. Utilizing this methodology, **35a** was coupled with two equiv of free cysteine in aqueous acetonitrile at 20 °C over 12 h to give bis(*S*-acylcysteine) **36** in 64% yield. Compound **36** was then treated with 1 equiv of **35a**–**c** to synthesize cyclic enantiopure peptidomimetic products **37a**–**c** in 81–82% yield (Scheme 2, see Supporting Information File 1 for experimental details).

**Scheme 3:**
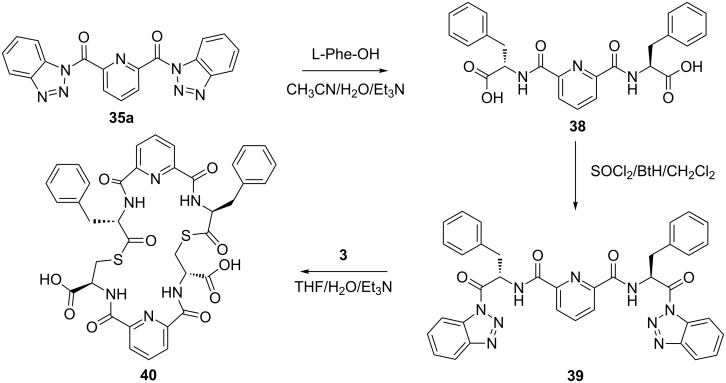
Preparation of pyridine–cysteine-containing macrocycle **39**.

In a further application of this synthetic approach **35** was coupled with 2 equiv of L*-*Phe-OH in the presence of TEA in aqueous acetonitrile at room temperature over 3 h giving the bis N-acylated compound **38** in 88% yield. Compound **38** was converted to the corresponding benzotriazole derivative **39** and coupled with bis(*S*-acylcysteine) **36** forming the pyridine–cysteine-containing macrocycle **40** in 70% yield (Scheme 3, see Supporting Information File 1 for experimental details).

### Bioassay

Screening for antibacterial activity was performed for two cyclic peptidomimetics belonging to scaffold **37**, namely **37a** and **37b**, with the pyridine- and phenyl-linking fragments, respectively. The array of microbial strains assayed in this study included six pathogens listed in Table 2 (see Supporting Information File 1, for antibacterial testing protocol).

**Table 2:** Antibacterial activity in vitro*.*

Microbial strain^a^	Inhibition zone (mm) at 200 µg/mL				MIC^b^ (µg/mL)				
		
	**37a**	**37b**	Rox^c^	Cfx^d^	**37a**		**37b**	Rox^c^	Cfx^d^

*Staphylococcus aureus* (ATCC 6538)	0.0	0.0	30	40	–		–	12.5	6.25
*Escherichia coli* (ATCC 8739)	0.0	0.0	14.6	22.9	–		–	100	50
*Bordetella bronchiseptica* (ATCC 4617)	0.0	6.0	8	33	–		150	150	12.5
*Micrococcus luteus* (ATCC 10240)	0.0	6.0	30	40	–		150	12.5	12.5
*Salmonella typhimurium* (ATCC 14028)	0.0	6.0	12	40	–		150	100	6.25
*Enterobacter aerogenes* (ATCC 13048)	0.0	0.0	15	34	–		–	100	12.5

^a^*Bordetella bronchistepica*: Gram negative, relevant to veterinary science; *Micrococcus luteus*: Gram positive, not dangerous for humans; *Salmonella typhimurium*: Gram negative, relevant to veterinary science. ^b^MIC = minimum inhibitory concentration. ^c^Rox = roxithromycin. ^d^Cfx = cefixime.

The in vitro data for **37a** and **37b** are given in Table 2. Reference antibiotics roxithromycin and cefixime were used as positive controls. It is seen that peptidomimetic **37b** exhibits moderate activity against *Bordetella bronchiseptica*, *Micrococcus luteus*, and *Salmonella typhimurium*. For a “bare” scaffold with no tailor-made substitution, this should be considered as an encouraging result and an indication of the possibility of more interesting results if the scaffold is furnished with appropriate functions.

Peptidomimetic **37a** showed no activity in this test, which can be explained by the formation of a zwitter-ionic structure due to protonation of the pyridine moiety by free carboxylic groups. It is reasonable to assume that such a zwitter-ionic structure alters the charge distribution and also eliminates hydrogen bonding in which the pyridine nitrogen atom acts as a H-bond acceptor. These factors increase hydrophobicity and thus deteriorate bioavailability, as reflected in the absence of activity of **37a**. Absence of data in Table 2 means that the sample is not active at the highest concentration tested.

## Conclusion

Two new cyclic peptidomimetic scaffolds were identified by using similarity-based rational design. Their chemical similarity with existing antibacterial cyclic peptidomimetics was established in a huge descriptor space generated by Codessa-Pro software. At least one compound (**37b**) demonstrated a moderate antibacterial activity against three bacterial strains, which is a fairly promising result for a “bare” cyclic scaffold with no intentional functionalization. Given the successful scaffold identified, the next step will be to furnish it with appropriate functional groups and substituents by using rational design principles.

## Experimental

### Methodology

#### Molecular similarity

The potential of cyclic peptidomimetics, widely acknowledged as significant, arises because they are less prone to hydrolysis in vivo and can be synthesized rather inexpensively. The synthetic methodology that has been developed in our lab affords facile assembles of cyclic structures from dicarboxylic building blocks by using a benzotriazole methodology. Seventeen and eighteen-membered sulfur-containing rings can be synthesized readily if cysteine is used as the coupling agent. Such facile access to cyclic peptidomimetics is appropriate for the design of compounds expected to be biologically active as antibacterial agents. Exploring the molecular similarity of existing antibacterial cyclic peptides using the Tanimoto method in the Instant JChem software [52], and the peptidomimetics easily accessible through the benzotriazole route, should give structural insights and design criteria. Guidance for such a similarity-based design should be available by analysis of a reasonably sized dataset of existing analogues. As abundant data can be found in the literature for *Staphylococcus aureus,* this was chosen as a reference for antibacterial activity. SciFinder Scholar was used to retrieve the structures of 33 cyclic peptides with reported antibacterial activity (Table 1). The minimum growth inhibition constant (MIC) was used as the measure of antibacterial activity, and the data collected were converted where necessary to μg/mL. Three structures newly synthesized in our lab were also added to the general dataset (Table 1). All these structures were drawn with the Marvin Beans Suite program [52] and preoptimized using the molecular mechanics utility (MM2) [53] in Chem3D Ultra 12.0 software [54]. Final geometry optimization of the compounds was carried out by using the semiempirical quantum-mechanical AM1 parameterization [55].

#### Molecular descriptors

The optimized geometries of the compounds were loaded into CODESSA Pro software [38]. Overall, more than 800 theoretical descriptors were calculated including constitutional, geometrical, topological, electrostatic, quantum-chemical, and thermodynamic molecular descriptors [56–57].

#### Cluster analysis

Cluster analysis of the descriptor hyperspace was performed by using STATISTICA version 6 software [58], with Euclidean distance and other metrics used as similarity measures. Cluster analysis guided by the experimental MIC available for some compounds should help reveal structural features affording antibacterial activity and to identify hotspots in the descriptor hyperspace. Combinations of structural features in such hotspots can be used as guidelines for the rational design of cyclic peptide structures to achieve desirable levels of antibacterial activity.

## Supporting Information

File 1 Experimental details, characterization data of synthesized compounds and antibacterial testing protocol.
