# Supplementary material for: Similarity analysis, synthesis, and bioassay of antibacterial cyclic peptidomimetics
Source: Beilstein J Org Chem. 2012 Jul 24;8:1146–60. doi: 10.3762/bjoc.8.128 (PMC3458733; doi:10.3762/bjoc.8.128)
Supplement: File 1 — Experimental details, characterization data of synthesized compounds and antibacterial testing protocol. [file Beilstein_J_Org_Chem-08-1146-s001.pdf]

Supporting Information
for
Similarity analysis, synthesis, and bioassay of antibacterial cyclic peptidomimetics

Workalemahu M. Berhanu[1] , Mohamed A. Ibrahim [1,2,3], Girinath G. Pillai[1], Alexander A. Oliferenko[1], Levan Khelashvili[1], Farukh Jabeen[4], Bushra Mirza[5], Farzana Latif Ansari[4], Ihsan ul-Haq[5], Said A. El-Feky[2], and Alan R. Katritzky*[1,6]

[1]Center for Heterocyclic Compounds, Department of Chemistry, University of Florida, Gainesville, FL 32611-7200, [2]Department of Pharmaceutical Organic Chemistry, Faculty of Pharmacy, Zagazig University, Zagazig-44519, Egypt, [3]Department of Organic Chemistry, College of Pharmacy, Misr University for Science and Technology, Al-Motamayez District, P.O. Box: 77, Egypt, [4]Department of Chemistry, Quaid i Azam University, Islamabad 45320, Pakistan, [5]Department of Biochemistry, Quaid i Azam University, Islamabad 45320, Pakistan and [6]Department of Chemistry, King Abdulaziz University, Jeddah 21589, Saudi Arabia

Email: Alan R. Katritzky* - katritzky@chem.ufl.edu

* Corresponding author

Experimental details, characterization data of synthesized compounds and antibacterial testing protocol.

Experimental details

Melting points were determined on a capillary point apparatus equipped with a digital thermometer and are uncorrected. The NMR spectra were recorded in $CDCl_3$, DMSO-d_6, CD_3OD, TFA-*d* with TMS for ^{1}H (300 MHz) and ^{13}C (75 MHz) as an internal reference. Elemental analysis was performed on a Carlo Erba EA 1108 elemental analyzer.

General procedure for the preparation of *N*-acyl benzotriazole derivatives (35a–c)

Thionyl chloride (0.95 mL, 13.17 mmol) was added to a solution of benzotriazole (5701 mg, 47.90 mmol) in dichloromethane (100 mL) and the solution was stirred at rt for 20 min. The dicarboxylic acids **34a–c** (6 mmol) were added to the mixture, which was then stirred at rt for 12 h. The precipitate was filtered off and the filtrate was extracted with saturated sodium carbonate solution (3 x 100 mL). The organic layer was dried over anhydrous sodium sulfate, filtered and evaporated under vacuum to give compounds **35a–c**.

Pyridine-2,6-diyl bis((1*H*-benzo[*d*][1,2,3]triazol-1-yl)methanone) (35a)

Yield: 54%; white microcrystals; mp 226–228 °C; ^{1}H NMR (DMSO-d_6) δ 7.70–7.88 (br s, 2H), 7.89–8.00 (br s, 2H), 8.33–8.49 (br s, 4H), 8.50–8.80 (br s, 3H); ^{13}C NMR (DMSO-d_6) δ 114.2, 120.2, 127.0, 128.9, 131.1, 131.3, 138.4, 145.2, 149.7, 164.2; Anal. calcd for $C_{19}H_{11}N_7O_2$: C, 61.79; H, 3.00; N, 26.55; found: C, 61.41; H, 2.94; N, 26.22.

1,3-Phenylenebis((1*H*-benzo[*d*][1,2,3]triazol-1-yl)methanone) (35b) [1]

Yield: 52%; white microcrystals; mp 189–191 °C. (lit. [1]: mp 189–191 °C); ^{1}H NMR ($CDCl_3$) δ 7.51 (t, *J* = 8.2 Hz, 2H), 7.64–7.78 (m, 3H), 8.11 (d, *J* = 8.4 Hz, 2H), 8.35 (d,

J = 8.4 Hz, 2H), 8.48 (dd, *J* = 7.8, 1.8 Hz, 2H), 8.98–9.00 (m, 1H); ^{13}C NMR ($CDCl_3$) δ 115.0, 120.5, 126.8, 128.9, 130.9, 132.2, 132.3, 135.2, 136.4, 146.0, 165.7.

1,4-Di(1*H*-benzo[*d*][1,2,3]triazol-1-yl)butane-1,4-dione (35c)

Yield: 37%; white microcrystals; mp 235–237 °C; ^{1}H NMR (DMSO-d_6) δ 4.02 (br s, 4H), 7.63 (t, *J* = 8.0 Hz, 2H), 7.80 (t, *J* = 7.5 Hz, 2H), 8.24 (d, *J* = 8.1 Hz, 2H), 8.29 (d, *J* = 8.4 Hz, 2H); Anal. calcd for $C_{16}H_{12}N_6O_2$: C, 60.00; H, 3.78; N, 26.24; found: C, 59.73; H, 3.73; N, 26.24.

(2*S*,2'*S*)-3,3'-[(pyridine-2,6-dicarbonyl)bis(sulfanediyl)]bis(2-aminopropanoic acid) (36).

L-Cysteine (290 mg, 2.39 mmol) was added to a solution of compound **34a** (400 mg, 1.05 mmol) in acetonitrile (25 mL) and water (6 mL). The mixture was stirred at rt for 12 h. The solid precipitate was filtered and washed with diethyl ether (2 x 50 mL). The white solid was dried under vacuum to complete dryness to give compound **36** (260 mg, 0.70 mmol) as microcrystals in 64% yield. Mp 236 °C; ^{1}H NMR (TFA–*d*) δ 3.63 (dd, *J* = 7.1, 5.0 Hz, 1H), 3.68 (dd, *J* = 7.1, 4.7 Hz, 1H), 3.85–3.97 (m, 2H), 4.69–4.79 (m, 2H), 8.05–8.21 (m, 3H); ^{13}C NMR (TFA–*d*) δ 30.3, 57.2, 128.0, 142.5, 152.3, 173.5, 197.9; Anal. calcd for $C_{13}H_{15}N_3O_6S_2$: C, 41.82; H, 4.05; N, 11.25; found: C, 41.50; H, 4.07; N, 11.08.

General Procedure for the preparation of the macrocycles (37a–c)

A solution of compound **3** (200 mg, 0.54 mmol) and triethylamine (0.30 mL, 2.15 mmol) in water (20 mL) was added dropwise to a solution of compound **35a–c** (0.54 mmol) in tetrahydrofuran (100 mL). The solution was stirred at rt for 3 h. The organic solvent was evaporated under reduced pressure and the aqueous layer was washed with 2 N HCl

and extracted with ethyl acetate (3 x 50 mL). The organic layer was dried over anhydrous sodium sulfate, filtered, and evaporated until complete dryness to give compounds **37a–c**.

2,7,13,18-Tetraoxo-3,17-dithia-6,14,23,24-tetraazatricyclo[17.3.1.18,12]tetracosa-1(23),8(24),9,11,19,21-hexaene-5,15-dicarboxyxlic acid (37a)

Yield : 81%; white microcrystals; mp 256–258 °C; ^{1}H NMR (DMSO-d_6) δ 3.53 (dd, J = 13.5, 5.4 Hz, 2H), 3.68 (dd, J = 13.5, 6.6 Hz, 2H), 4.80–4.87 (m, 2H), 8.13–8.31 (m, 6H), 9.36 (d, J = 8.1 Hz, 2H); ^{13}C NMR (DMSO-d_6) δ 29.3, 51.2, 123.5, 124.9, 139.7, 141.0, 149.2, 149.5, 163.7, 170.9, 191.4; Anal. calcd for $C_{20}H_{16}N_4O_8S_8 \cdot H_2O$: C, 45.97; H, 3.09; N, 10.72; found: C, 45.70; H, 3.29; N, 10.23.

2,7,13,18-tetraoxo-6,14-dithia-3,17,23-triazatricyclo[17.3.1.$1^{8,12}$]tetracosa-1(23),8(24),9,11,19,21-hexaene-4,16-dicarboxylic acid (37b)

Yield: 81%; white microcrystals; mp 230 °C; ^{1}H NMR (CD_3OD) δ 3.66 (dd, J = 14.3, 7.1 Hz, 2H), 3.88 (dd, J = 14.4, 4.5 Hz, 2H), 4.82–5.16 (m, 2H), 7.53–7.63 (m, 1H), 7.92–7.96 (m, 1H), 8.09–8.28 (m, 4H), 8.64–8.70 (m, 1H); ^{13}C NMR (CD_3OD) δ 32.1, 53.0, 126.5, 130.9, 131.6, 131.9, 139.8, 140.4, 150.9, 166.2, 172.9, 193.2; Anal. calcd for $C_{21}H_{17}N_3O_8S_2 \cdot 3H_2O$: C, 45.24; H, 4.16; N, 7.54; found: C, 44.69; H, 4.00; N, 7.23.

2,7,10,15-tetraoxo-6,11-dithia-3,14,20-triazabicyclo[14.3.1]icosa-1(20),16,18-triene-4,13-dicarboxylic acid (37c)

Yield: 82%; white microcrystals; mp 83–85 °C; ^{1}H NMR (DMSO-d_6) δ 2.78–2.87 (m, 2H), 3.03–3.16 (m, 2H), 3.37 (dd, J = 14.1, 3.3 Hz, 2H), 3.58 (dd, J = 14.1, 9.9 Hz, 2H), 4.51–4.59 (m, 2H), 8.10–8.30 (m, 3H), 9.33–9.41 (m, 2H), 12.95 (br s, 2H); ^{13}C NMR

(DMSO-d_6) δ 29.5, 39.1, 52.9, 125.1, 140.0, 148.8, 163.8, 171.8, 197.6; Anal. calcd for $C_{17}H_{17}N_3O_8S_2 \cdot H_2O$: C, 43.12; H, 4.04; N, 8.87; found: C, 42.85 ; H, 4.23; N, 8.32.

***N*^{2},*N*^{6}-bis(3-phenylpropanoic acid-2-yl)pyridine-2,6-dicarboxamide (38)** [2]

A solution of phenylalanine (985 mg, 5.96 mmol) and triethylamine (1.5 mL, 10.84 mmol) in water (150 mL) was added portionwise to a solution of compound **34** (1000 mg, 2.71 mmol) in acetonitrile (500 mL). The solution was stirred at rt for 3 h. The solvent was evaporated under vacuum. The concentrated solution was diluted with ethyl acetate (100 mL) and washed with 2 N HCl (3 x 75 mL). The organic layer was dried over anhydrous sodium sulfate, filtered and evaporated under reduced pressure to complete dryness to give compound **38** (1100 mg, 2.38 mmol) in 88% yield. Mp 76–78 °C; ^{1}H NMR (DMSO-d_6) δ 4.31–4.58 (m, 4H), 5.76–5.96 (br s, 2H), 8.20–8.58 (m, 10H), 9.18–9.43 (m, 3H), 10.43–10.46 (d, J = 6.9 Hz, 2H), 13.85–14.28 (br s, 2H); ^{13}C NMR (DMSO-d_6) δ 36.4, 54.2, 124.7, 126.4, 128.3, 129.1, 138.0, 139.5, 148.3, 163.1, 172.6.

***N*^{2},*N*^{6}-Bis(1-(1*H*-benzo[*d*][1,2,3]triazol-1-yl)-1-oxo-3-phenylpropan-2-yl)pyridine-2,6-di caboxamide (39)**

Thionyl chloride (0.15 mL, 2.14 mmol) was added to a solution of benzotriazole (928 mg, 7.80 mmol) in dichloromethane (100 mL). The solution was stirred for 30 min at rt, then compound **37** (450 mg, 0.975 mmol) was added. The suspension was stirred at rt for 6 h. The precipitate was filtered and the filtrate was extracted with a saturated sodium carbonate aqueous solution (3 x 100 mL). The organic layer was dried over anhydrous sodium sulfate and evaporated under vacuum to complete dryness to give compound **39** (400 mg, 0.60 mmol) in 62% yield. Mp 160 °C; ^{1}H NMR($CDCl_3$) δ 3.39–

3.53 (m, 2H), 3.65–3.74 (m, 2H), 6.42–6.55 (m, 2H), 7.00–8.62 (m, 23H); ^{13}C NMR ($CDCl_3$) δ 39.0, 54.8, 114.5, 120.7, 125.9, 126.9, 127.8, 129.2, 129.6, 131.2, 135.4, 139.4, 146.3, 148.3, 163.5, 170.5.

8,18-dibenzyl-2,7,10,16,19,24-hexaoxo-6,20-dithia-3,9,17,23,29,30-hexaazatricyclo [23.3.1.1^{11,15}]triaconta-1(29),11(30),12,14,25,27-hexaene-4,22-dicarboxylic acid (40)

A solution of compound **36** (200 mg, 0.54 mmol) and triethylamine (0.244 mL, 1.61 mmol) in water (20 mL) was added to a solution of compound **39** (355 mg, 0.536 mmol) in acetonitrile (100 mL). The solution was stirred at rt for 14 h, concentrated under reduced pressure, washed with 2 N HCl and extracted with ethyl acetate (3 x 100 mL). The organic layer was dried over anhydrous sodium sulfate, filtered and evaporated to give an oily compound. Trituration of it with diethyl ether gave a yellow solid, which was dried under vacuum to give compound **40** (300 mg, 0.37 mmol) in 70% yield. Mp 110 °C; ^{1}H NMR (CD_3OD) δ 3.03–3.79 (m, 6H), 4.24–5.11 (m, 6H), 6.93–7.60 (m, 10H), 7.99–8.34 (m, 8H), 8.71–9.47 (br s, 1H), 9.60–10.10 (br s, 2H); ^{13}C NMR(CD_3OD) δ 26.8, 31.0, 53.7, 63.7, 126.3, 126.5, 127.7, 127.9, 129.7, 130.1, 130.4, 138.8, 140.7, 149.6, 165.7, 166.0, 173.1, 200.4; Anal. calcd for $C_{38}H_{38}N_6O_{12}S_2{\cdot}2H_2O$: C, 54.67; H, 4.10; N, 10.07; found: C, 54.56 ; H, 4.35; N, 9.49.

Antibacterial testing protocol

For the determination of antibacterial activity of the test samples, a disc diffusion method was employed as described by Klancnik et al [3]. Briefly, two petri plates (7 cm) containing 20 mL of nutrient agar seeded with 1% v/v inoculum of 24 h old bacterial cultures having turbidity adjusted to McFarland turbidity standard 0.5 were prepared for each bacterial strain. Five sterile paper discs (4 cm diameter) impregnated with 5 µL of

test sample or standard solution containing 200 μg/5μL of test sample were place on each plate and incubated at 37 °C for 24 h. The test was performed in duplicate, cefixime and roxithromycin were used as standard antibacterial drugs and DMSO was used as a negative control. Cefixime and roxithromycin have diverse mechanisms of action, i.e., inhibition of cell-wall synthesis and protein synthesis, respectively. We have used different Gram positive and Gram negative bacteria in this study, and these bacteria have different sensitivity to different antibiotics. Thus, it was better to use two different antibiotics as positive controls rather than a single. Roxithromycin shows stronger activity against Gram positive bacteria and cefixime is more effective against Gram negative bacteria. After 24 h of incubation, a clear zone of inhibition around each disc was noted. The compounds that showed antibacterial activity in the disc-diffusion test were selected to determine their MICs (minimum inhibitory concentrations) by using a broth microdilution method as also described by Klancnik et al [3]. In this assay sterile 96-well plates were used and six concentrations (200, 150, 100, 50, 25 and 12.5 μg/mL) were tested to find the MIC. To each well, 95 μL of the bacterial culture having turbidity adjusted to McFarland turbidity standard 0.5 was added and 5 μL of the test compound solution in DMSO was added to get the final concentration as described above. Plates were covered and mixed on rotary shaker at 500 rpm for 2 min and then incubated at 37 °C for 24 h. After incubation, plates were taken out and 10 μL of 20 mg/mL solution of TTC (3,4,5-triphenyl tetrazolium chloride) in sterile distilled water was added to each well and mixed on rotary shaker for 2 min and again incubated for 20 min. After 20 min, the lowest concentration for which the compound did not develop a red color was taken as the MIC.

References

1. Lu, C. X.; Li, Q.; Pan, J. *J. Polymer Sc. Polymer Chem. Ed.* **1985**, *23*, 3031–3044.
2. Attia, A.; El-Salam, O. I. A.; Amr, A.; Stibor, I.; Budesinky, M. *Egyptian J. Chem.* **2000**, *43*, 187–201.
3. Klancnik, A.; Piskernik, S.; Jersek, B.; Mozina, S. S. *J. Microbiol. Meth.* **2010**, 81, 121–126.
